# Frequency and reasons for delayed treatment initiation after HIV diagnosis: cross-sectional study in Lahore, Pakistan

**DOI:** 10.1186/s12889-021-11031-0

**Published:** 2021-05-27

**Authors:** Hassan Ali, Rubeena Zakar, Khunsa Junaid, Amjad Khan, Florian Fischer

**Affiliations:** 1 Department of Public Health, Institute of Social and Cultural Studies, University of the Punjab, Lahore, Pakistan; 2 Department of Public Health, The University of Haripur, Haripur, Pakistan; 3 Institute of Public Health, Charité, Universitätsmedizin Berlin, Charitéplatz 1, 10117 Berlin, Germany; 4 Institute of Gerontological Health Services and Nursing Research, Ravensburg-Weingarten University of Applied Sciences, Weingarten, Germany

**Keywords:** HIV, Delayed initiation, ART, PCR

## Abstract

**Background:**

Well-timed initiation of HIV therapy enhances life expectancy, decreases mortality and morbidity, and inhibits the transmission of HIV and complications related to it. The purpose of the present survey is to investigate the frequency and reasons for delayed initiation of anti-retroviral therapy (ART) and to determine its relationship with various socio-demographic variables and HIV-related characteristics.

**Methods:**

The analysis is based on a cross-sectional study involving 355 people living with HIV (diagnosed by PCR) who were more than 18 years of age and not receiving HIV therapy before enrolment at the HIV clinics of two selected tertiary-care teaching hospitals in Lahore, Pakistan. In this study, delayed initiation of ART was defined as not attending the HIV management centre or a clinic for ART within 3 months of a confirmed diagnosis. The participants were selected using a systematic probability sampling technique. Bivariate logistic regression was performed using a backward stepwise technique to establish the variables related to delayed onset of HIV therapy. Factors significant at *p* ≤ 0.20 were considered for multivariate analysis, which was used to describe the association between independent factors and delayed initiation of treatment.

**Results:**

Delayed onset of ART was observed in 28.5% of individuals. Factors such as no schooling (AOR = 5.92; 95% CI: 1.38–25.41; *p* = 0.017) and occasional household income (AOR = 3.88; 95% CI: 1.01–14.89; *p* = 0.048) were significantly associated with late onset of ART. Our research findings also indicated that the main reasons for late beginning of HIV therapy were: feeling healthy (45.5%), did not have time to go to the HIV treatment centre (42.6%), did not want to discuss HIV test result (37.6%), and fear of stigma and discrimination within their community (35.6%).

**Conclusions:**

Late commencement of HIV therapy in Pakistan is common, and an improved connection is needed between identification of HIV and beginning of therapy. HIV management centres should counsel and monitor patients from the time of a positive HIV test result until they initiate therapy.

**Supplementary Information:**

The online version contains supplementary material available at 10.1186/s12889-021-11031-0.

## Background

The human immunodeficiency virus (HIV) still results in a substantial number of deaths every year due to acquired immunodeficiency syndrome (AIDS)-related illnesses. Globally, there are about 38 million people living with HIV (PLHIV), and approximately 690,000 individuals died from AIDS-related disorders in 2019 [1]. In 2019, about 190,000 PLHIV were present in Pakistan, with a prevalence of 0.1% in adults aged 15–49 years [2].

Numerous socioeconomic factors, including poverty, lack of education, and unemployment, are likely to promote the transmission of HIV in Pakistan [3]. PLHIV exist in almost all population sub-groups. For example, HIV outbreaks comprising mainly male sex workers (MSWs) are expanding significantly in comparison with female sex workers (FSWs), revealing diversity in localized sex practices [4, 5]. HIV outbreaks are rapidly increasing among intravenous drug users (IDUs). IDUs having sexual contacts with MSWs and transgendered individuals are the main reason for HIV escalation in many areas of Pakistan. As a consequence of this, Pakistan appears to be following the “Asian Epidemic Model” [5].

The majority of PLHIV who do not receive HIV treatment are reported to have a recognizable virus load in their bodily fluids, and, therefore, may spread the virus to their sexual partners. According to prevention policies related to HIV/AIDS, early detection and commencement of anti-retroviral treatment (ART) can remarkably reduce disease transmission [6–8]. Late commencement of ART results in increased admissions to hospital owing to opportunistic infections [6, 7]. Timely initiation of ART and management of opportunistic infections will reduce mortality and morbidity [7, 8], and will prevent secondary transmission and HIV-associated complications. Therefore, it will result in enhanced health and life expectancy for PLHIV [8–11].

Ideally, ART should be started before the beginning of opportunistic infections. However, both in economically developed and developing nations, many PLHIV start HIV treatment late [12]. The primary reasons for the late onset of HIV treatment vary in relation to community and local traditions [6, 8]. One of the reasons for late onset of HIV treatment is lack of awareness about the World Health Organization’s (WHO) guidelines on the timing of ART [11]. According to WHO guidelines on HIV treatment, in order to prevent HIV transmission, early initiation of ART is recommended irrespective of CD4 count [13]. Nevertheless, in Pakistan the old guidelines are still followed for use of ART [14]. According to these guidelines, ART treatment can be initiated in all PLHIV presenting with clinical symptomatic disease or asymptomatic disease with total lymphocyte count (TLC) below 1200/mm^3^, if a CD4 assay is not available. But in cases where a CD4 assay is available, ART will be started for WHO stage IV disease (when PLHIV develop AIDS) regardless of CD4 count, or WHO stage III disease (presence of symptoms due to opportunistic infections) with CD4 count below 350 cells/mm^3^, or asymptomatic disease with CD4 count below 200 cells/mm^3^ [14]. In Pakistan, only 10% of PLHIV are getting ART, and adherence to treatment is also very low [15].

Until now, evidence about the reasons for late onset of HIV treatment from economically developing countries outside the African continent has been lacking and there is no study available in Pakistan to address this issue. Therefore, the present study aims to identify the frequency and reasons for delayed initiation of HIV treatment in Pakistan and determine its association with socio-demographic variables and HIV-related characteristics.

## Methods

### Study design

The cross-sectional study was conducted between December 2017 and November 2018 in the HIV clinics of two hospitals in Lahore, Pakistan, which are the largest teaching hospitals in the public sector. These HIV clinics work under the administrative control of the Punjab AIDS Control Programme (PACP). The city of Lahore was selected for this research because of its diverse socio-demographic and economic profile. The Integrated Behaviour and Biological Survey 2011–2012 indicated a high prevalence of HIV in several cities in Pakistan. For example, in Lahore the HIV prevalence was 30.8% among people who inject drugs, 5.2% among transgender (*hijra*) sex workers, 1.7% among male sex workers, and 0.5% among female sex workers [16]. Another reason for the selection of Lahore was that it is the capital of Punjab Province of Pakistan. Most of the HIV clinics in Punjab are situated in Lahore. Due to this, PLHIV across Punjab come to Lahore for their treatment.

Careful methodological and ethical considerations (such as informed consent, voluntary participation, privacy, confidentiality, and anonymity) are necessary when performing research on sensitive topics, particularly in countries like Pakistan. Considering this, data from PLHIV were collected at the HIV clinics of two selected teaching hospitals of Lahore. Pakistan is a traditionalist country, where performing research on sensitive issues like HIV requires careful consideration of ethical issues. HIV clinics are situated in teaching hospitals and interviews were carried out in these hospitals in a separate room attached to the HIV clinic where they could proceed without any interference from outsiders or family members of patients. In the Pakistani family set up, it is difficult to interview an HIV patient alone in a household setting. Additionally, the presence of others may prevent respondents from giving accurate information about their disease. There is also a real risk that the questions relating to HIV that are asked during the interview may offend relatives of the respondents.

Confidentiality, anonymity, and privacy for all study participants were guaranteed at all levels in this study. No identifying information about the patient, such as name, date of birth, telephone number, or address, was recorded. Verbal informed consent was taken from each patient before they enrolled in the study. The aim of the research was explained to the patients in detail. Patients were also informed about the possible risks/discomfort involved. The data collection was carried out in a comfortable environment in a separate room attached to the HIV clinic where patients had the opportunity to speak openly, and they also had the right to ask any question about the study. All aspects of data collection were carried out in accordance with the “Guidelines and Recommendations to Assure Good Epidemiologic Practice” developed by the German Society for Epidemiology [13].

### Respondents

We recruited PLHIV, including both men and women who had not received HIV treatment before their enrolment at the HIV clinics of two selected hospitals in Lahore during the study period. Only HIV cases diagnosed by polymerase chain reaction (PCR) performed at a standardized laboratory were included. Patients younger than 18 years of age were not included in this study. Respondents who were not willing to participate and those who had started HIV treatment before enrolling at the HIV clinic of the selected hospitals were also excluded.

### Sampling

Taking 30% as the proportion of late onset of HIV treatment [12], the sample size was determined by applying the following formula, using a 5% margin of error with a 95% confidence interval (CI): $\mathrm{n}=\frac{{\mathrm{z}}^2\mathrm{pq}}{{\mathrm{d}}^2}$ [17]. With these parameters, the calculated sample size was 323, which was then increased by 10% to allow for an anticipated non-response rate. Therefore, in total, 355 PLHIV were included in this study.

The study respondents were selected using a systematic probability sampling technique. About 1000 new PLHIV visit the HIV clinic every year to start HIV treatment. Based on this information, we obtained the k-value: $\mathrm{k}=\frac{N}{n}=\frac{\ (1000)}{(355)}$ ≌ 3. Based on this result, we took the second patient from the first group of three patients in the list as our first respondent, and then every third patient afterwards until we achieved the required sample size.

### Measures

Data were assessed using a structured questionnaire (see Supplementary Appendix) in face-to-face interviews. The dependent variable was ‘delay in initiation of HIV treatment’. The delay was defined according to the Centres for Disease Control and Prevention (CDC) directions as PLHIV who did not visit the HIV management centre for HIV treatment within 3 months of a confirmed diagnosis [18].

The independent variables encompassed socio-demographic factors (age, gender, religion [Muslim vs. Christian], place of residence, marital status, education, employment [employed vs. unemployed], and type of household income [permanent vs. occasional; where occasional household income refers to members of a household earning money occasionally or sometimes but not regularly. This occurs when they do not have a regular source of income. They work for daily wages and are sometimes unable to find work and do not earn money.]) and HIV-related characteristics (initial CD4 count, physical disability, and associated chronic diseases).

The questionnaire was developed on the basis of an extensive literature review [6, 9, 11, 12] and informal discussions with health experts managing HIV cases in Lahore. Questions were asked about HIV testing conducted due to an HIV-positive partner, due to the appearance of symptoms, as a routine test during pregnancy or before any surgery, or due to other reasons, in order to collect information regarding the reasons for HIV testing.

Furthermore, we assessed the reasons for delay in the initiation of HIV treatment. We asked questions in relation to different reasons, such as feeling healthy and judging the treatment as unnecessary, fear-related reasons (e.G. *stigma* and discrimination in the community or at the facility), time constraints making it difficult to visit the health facility, fear of the side effects of medication, insufficient knowledge about the HIV treatment facilities, inadequate accessibility, fear that HIV treatment is not confidential, concerns related to costs and having no health insurance, fear of detention or imprisonment, and perceptions regarding the low quality of service at the facility.

### Statistical analysis

Data was analysed using SPSS version 26.0. After performing descriptive statistics, a chi-square test was applied. In addition, a bivariate logistic regression was performed using a backward stepwise technique to establish the variables related to delayed HIV treatment. Factors that were significant at *p* < 0.20 were considered for the multivariate model [19]. The strengths of association were evaluated for factors related to late commencement of ART by means of adjusted odds ratios (AORs) with 95% confidence intervals (CIs). The Hosmer-Lemeshow test was applied to analyse the goodness of fit for the final model [20].

## Results

### Socio-demographic characteristics

In total, 355 PLHIV (diagnosis confirmed by PCR test) were enrolled in this survey. The mean age of respondents was 31.75 ± 8.53 years. Among these, 261 (73.5%) were men, 74 (20.9%) were women and 20 (5.6%) were transgender. With respect to religion, 339 (95.5%) were Muslims and 16 (4.5%) were Christians. Just under half, 166 (46.8%), were residents of district Lahore. Most of the study respondents were living in urban areas (81.4%) and married (60.3%). About one third (32.4%) had no formal schooling. About half of the study respondents (53.8%) were employed. A little over half (59.4%) had a regular source of income, whereas 144 (40.6%) had only a temporary source of income (i.e., daily-waged jobs such as laborers, vendors, or salesmen) (Table 1).

**Table 1:** Socio-demographic characteristics of People living with HIV

Variables	*n*	%
**Age (in years)**		
18–29	162	45.6
30–39	124	34.9
40 and above	69	19.5
Mean ± SD	31.75 ± 8.53	
**Gender**		
Male	261	73.5
Female	74	20.8
Transgender	20	5.6
**Religion**		
Muslim	339	95.5
Christian	16	4.5
**District**		
Lahore	166	46.8
Other	189	53.2
**Place of residence**		
Urban	289	81.4
Rural	66	18.6
**Marital status**		
Unmarried	119	33.5
Married	214	60.3
Separated	5	1.4
Divorced	3	0.8
Widowed	14	3.9
**Educational level**		
No schooling	115	32.4
Primary	82	23.1
Secondary	99	27.9
College	39	11.0
University	20	5.6
**Employment status**		
Employed	191	53.8
Unemployed	164	46.2
**Type of household income**		
Permanent income	211	59.4
Occasional income	144	40.6

### HIV-related characteristics

About 72% (254) of respondents had started ART within 3 months of their HIV diagnosis. However, this also implies that 28% of HIV-diagnosed individuals had started ART late. About half of the study respondents (49.9%) had greater than 349 cells/mm^3^ initial CD4 count. Most had no physical disability (82.5%), while post-HIV and pre-HIV physical disability was present in 15.2 and 2.3%, respectively. In terms of chronic diseases, 23.7% had hepatitis C, 15.5% had tuberculosis, 2.3% had diabetes, and 2.0% had hepatitis B (Table 2).

**Table 2:** HIV-related and chronic disease related characteristics of people living with HIV

Variables	*n*	%
**Initiation of HIV treatment**		
Early (≤3 months)	254	71.5
Delayed (> 3 months)	101	28.5
**Initial CD4 count (cells/mm**^**3**^**)**		
< 200	105	29.6
200–349	76	21.4
> 349	174	49.0
Median ± IQR	341 ± 170	
**Physical disability**		
No	293	82.5
Pre-HIV	8	2.3
Post-HIV	54	15.2
**Any associated chronic disease**		
Tuberculosis	55	15.5
Diabetes	8	2.3
Hypertension	4	1.1
Hepatitis B	7	2.0
Hepatitis C	84	23.7
None	197	55.5

**Fig. 1:**
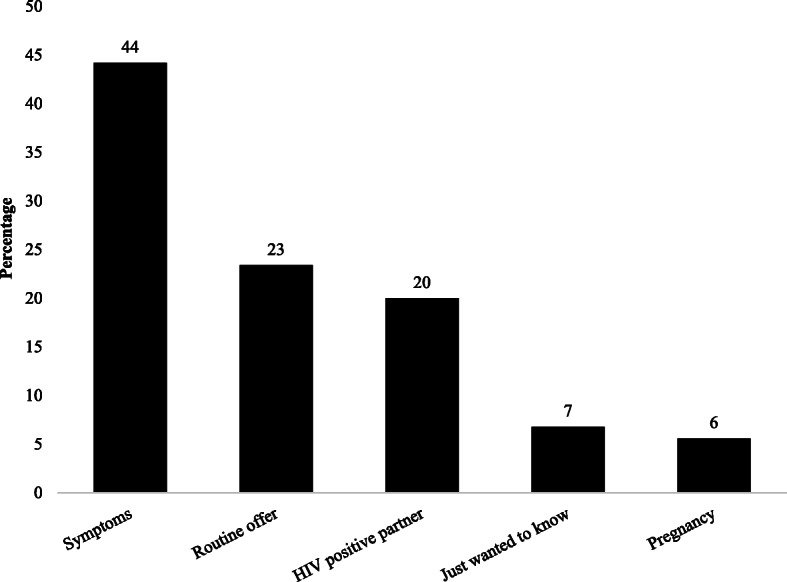
Reason for HIV testing among people living with HIV (*N* = 355)

**Fig. 2:**
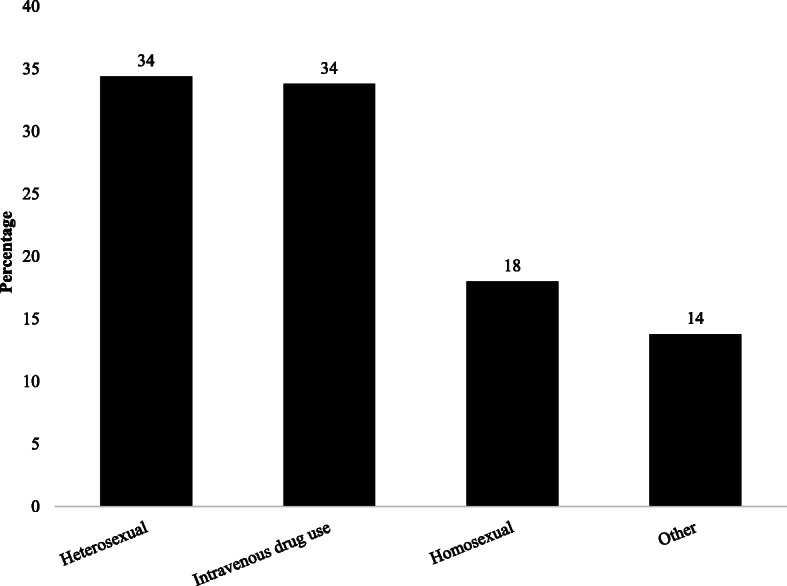
Risk factor for HIV transmission among people living with HIV (*n* = 355)

HIV testing was carried out due to the appearance of symptoms in 44.2% of cases, as part of routine follow-up in 23.4% of cases, due to a partner living with HIV in 20.0% of cases, as a routine test during pregnancy in 5.6% of cases, and just to check HIV status in 6.8% of cases (Fig. 1). Heterosexuality was reported by 122 (34.4%) respondents as a risk factor for transmission of HIV, IDU by 120 (33.8%) respondents, and homosexuality by 64 (18.0%) respondents (Fig. 2).

### Reasons for delayed onset of HIV treatment

This study found that the main reasons for delayed initiation of ART were that patients felt healthy (45.5%), did not have time to go to the HIV treatment centre (42.6%), did not want to discuss their HIV test result (37.6%), or were afraid of stigma and discrimination within their community (35.6%) (Table 3).

**Table 3:** Reasons for delay in initiation of HIV treatment (*n* = 101)

Reasons	*n*	%
Felt healthy and thought the treatment was not necessary	46	45.5
Fear of stigma and discrimination in community	36	35.6
Did not have time to go to HIV treatment centre	43	42.6
Fear the HIV treatment is not confidential	30	29.7
Fear of medication side effects	31	30.7
Afraid of the cost / Did not have money	26	25.7
Fear of stigma and discrimination at facility	25	24.8
Did not want to take medicine for HIV	34	33.7
Did not know the location of HIV treatment centre	33	32.7
HIV treatment centre too far away	29	28.7
Administration and formalities are too difficult	11	10.9
Detention or imprisonment	5	5.0
Fear of detention or imprisonment	14	13.9
Perception of low service quality at facility	13	12.9
Did not want to think about being HIV-positive	32	31.7
Didn’t believe in HIV test result	34	33.7
Did not want to discuss HIV result	38	37.6
Did not have an insurance	7	6.9
Moved out of town	6	5.9
Test not performed in the hospital providing HIV care	31	30.7

### Bivariate analyses

Our study results revealed that the socio-demographic variables of gender (χ^2^ = 8.622; *p* = 0.013), place of residence (χ^2^ = 6.182; *p* = 0.013), level of education (χ^2^ = 20.580; *p* < 0.001), employment status (χ^2^ = 7.161; *p* = 0.007), and type of household income (χ^2^ = 11.300; *p* = 0.001) were significantly associated with late commencement of ART (Table 4). With respect to HIV-related characteristics, initial CD4 count (χ^2^ = 6.652; *p* = 0.036) was seen to be significantly associated with late commencement of ART (Table 5).

**Table 4:** Association between late commencement of HIV treatment and socio-demographic factors

Variables	Initiation of HIV treatment		Total *n* (%)	χ^**2**^-value	***P***-value
	Delayed (***n*** = 101)	Early (***n*** = 254)			
	%	%			
**Age (in years)**					
18–29	48.5	44.5	162 (45.6)	2.627	0.269
30–39	28.7	37.4	124 (34.9)		
40 and above	22.8	18.1	69 (19.5)		
**Gender**					
Male	73.3	73.6	261 (73.6)	8.622	0.013
Female	15.8	22.8	74 (20.8)		
Transgender	10.9	3.6	20 (5.6)		
**Religion**					
Muslim	94.1	96.1	339 (95.5)	0.674	0.412
Christian	5.9	3.9	16 (4.5)		
**District**					
Lahore	39.6	49.6	166 (46.8)	2.904	0.088
Other	60.4	50.4	189 (53.2)		
**Place of residence**					
Urban	73.3	84.6	289 (81.4)	6.182	0.013
Rural	26.7	15.4	66 (18.6)		
**Marital status**					
Unmarried	32.7	33.9	119 (33.5)	0.269	0.992
Married	61.3	59.8	214 (60.3)		
Separated	1.0	1.6	5 (1.4)		
Divorced	1.0	0.8	3 (0.8)		
Widowed	4.0	3.9	14 (3.9)		
**Educational level**					
No schooling	47.5	26.4	115 (32.4)	20.580	< 0.001
Primary	25.7	22.0	82 (23.1)		
Secondary	17.8	31.9	99 (27.9)		
College	5.9	13.0	39 (11.0)		
University	3.1	6.7	20 (5.6)		
**Employment status**					
Employed	42.6	58.3	191 (53.8)	7.161	0.007
Unemployed	57.4	41.7	164 (46.2)		
**Type of household income**					
Permanent income	45.5	65.0	211 (59.4)	11.300	0.001
Occasional income	54.5	35.0	144 (40.6)

**Table 5:** Association between late commencement of HIV treatment and HIV and other related characteristics

Variables	Initiation of HIV treatment		Total n (%)	χ^**2**^-value	***p***-value
	Delayed (***n*** = 101)	Early (***n*** = 254)			
	%	%			
**Initial CD4 count (cells/mm**^**3**^**)**					
< 200	19.8	33.5	105 (29.6)	6.652	0.036
200–349	25.7	19.6	76 (21.4)		
> 349	54.5	46.9	174 (49.0)		
**Reason for HIV testing**					
Appearance of symptoms	38.6	46.5	157 (44.2)	6.012	0.198
HIV positive partner	19.8	20.1	71 (20.0)		
Routine test	31.7	20.0	83 (23.4)		
Pregnancy	4.0	6.3	20 (5.7)		
Just wanted to know	5.9	7.1	24 (6.8)		
**Risk factor for HIV transmission**					
Heterosexual	28.7	36.6	122 (34.4)	7.382	0.061
Homosexual	15.8	18.9	64 (18.0)		
IDU	44.6	29.5	120 (33.8)		
Other	10.9	15.0	49 (13.8)		
**Physical disability**					
No	82.2	82.7	293 (82.5)	0.286	0.958
Pre-HIV	2.0	2.3	8 (2.3)		
Post-HIV	15.8	15.0	54 (15.2)		
**Any associated chronic disease**					
Tuberculosis	14.9	15.7	55 (15.5)	8.209	0.145
Diabetes	3.0	2.0	8 (2.3)		
Hypertension	1.0	1.2	4 (1.1)		
Hepatitis B	5.0	0.8	7 (2.0)		
Hepatitis C	26.7	22.4	84 (23.7)		
None	49.4	57.9	197 (55.5)		

### Logistic regression

We applied a bivariate logistic regression to investigate the independent predictors related to late commencement of HIV treatment. All factors that were significant at *p* < 0.20 in the bivariate regression analysis, consisting of district, place of residence, educational level, employment status, household income, initial CD4 count, reason for HIV testing, and any associated chronic disease, were included in a multivariable logistic regression model. After applying multivariate regression, the factors: district other than Lahore, living in a rural area, no formal schooling, occasional income, and greater than 349 cells/mm^3^ initial CD4 count were seen to be significantly associated with delayed onset of HIV treatment (*p* < 0.05). After assessing the magnitudes of these associations, the results indicate that the respondents residing in district Lahore were less likely to be late for HIV treatment than residents of other districts (AOR = 0.52; 95% CI: 0.31–0.87), and respondents residing in cities had 0.45 times lower chances of late HIV treatment initiation than those residing in rural areas (AOR = 0.45; 95% CI: 0.24–0.83). Respondents having no formal schooling were more likely to have delayed treatment initiation than university graduates (AOR = 5.92; 95% CI: 1.38–25.41). Respondents with a temporary source of income had greater chances of being late for HIV treatment than those with a permanent income (AOR = 3.88; 95% CI: 1.01–14.89). The study also concluded that respondents having a CD4 count of fewer than 200 cells/mm^3^ had 0.48 times lower chances of being late for HIV treatment than those having a CD4 count of more than 349 cells/mm^3^ (AOR = 0.48; 95% CI: 0.25–0.93) (Table 6).

**Table 6:** Association between socio-demographic and HIV related factors and delayed onset of HIV treatment among HIV patients

Variables	OR^a^	95% CI		*P*-value	AOR^b^	95% CI		*P*-value
		Lower	Upper			Lower	Upper	
**District**								
Lahore	0.666	0.417	1.064	0.089	0.518	0.308	0.872	0.013
Other	1				1			
**Place of residence**								
Urban	0.497	0.285	0.868	0.014	0.446	0.239	0.831	0.011
Rural	1				1			
**Educational level**								
No schooling	4.060	1.126	14.632	0.032	5.916	1.378	25.408	0.017
Primary	2.631	0.708	9.776	0.149	3.576	0.881	14.519	0.075
Secondary	1.259	0.333	4.758	0.734	1.609	0.401	6.447	0.502
College	1.030	0.229	4.638	0.969	1.329	0.272	6.488	0.725
University	1				1			
**Employment status**								
Employed	1				1			
Unemployed	1.883	1.181	3.003	0.008	0.257	0.062	1.065	0.061
**Type of household income**								
Permanent income	1				1			
Occasional income	2.217	1.387	3.542	0.001	3.885	1.014	14.889	0.048
**Initial CD4 count ** (cells/mm)^3^								
< 200	0.509	0.284	0.912	0.023	0.479	0.248	0.925	0.028
200–349	1.125	0.635	1.993	0.686	1.326	0.707	2.486	0.380
> 349	1				1			
**Reason for HIV testing**								
Symptoms	0.992	0.368	2.675	0.987	0.900	0.302	2.684	0.849
HIV-positive partner	1.176	0.408	3.392	0.764	1.255	0.393	4.007	0.702
Routine offer	1.882	0.676	5.242	0.196	2.281	0.742	7.008	0.150
Pregnancy	0.750	0.179	3.144	0.694	0.692	0.143	3.351	0.647
Just wanted to know	1				1			
**Any associated chronic disease**								
Present	1.401	0.882	2.226	0.153	1.590	0.949	2.665	0.078
Absent	1				1			

1 = reference category

^a^*OR* Odds ratio

^b^*AOR* Adjusted odds ratio. Results obtained from final model after applying backward stepwise multivariable logistic regression

## Discussion

This study has highlighted the frequency and reasons for delayed onset of HIV treatment in Lahore, Pakistan, and determined the relationship between delayed treatment onset and both socio-demographic and HIV-related characteristics. The characteristics of HIV-positive individuals within our study regarding risk factors for delayed initiation of HIV treatment are comparable to previous research conducted in other countries, including Brazil [21], Vietnam [6], and the United States of America (USA) [10]. This also applies to the presence of tuberculosis and other chronic diseases, such as hepatitis B and C, which were associated with an HIV infection. This was also observed in our study, where a large proportion of the respondents (44.2%) were tested for HIV after the appearance of symptoms. These findings are consistent with previous research conducted in India [12, 22].

Our study results show that the prevalence of late commencement of HIV treatment in Pakistan is about 28.5%. This urgently needs to be addressed. Overall, this proportion of late onset of HIV treatment is comparable to studies conducted in Kenya [23], India [12], and the USA [10]. Previous studies from Brazil [21] and Cameroon [24] have revealed a relatively lower percentage of late onset of treatment; i.e., 9 and 15% respectively. Furthermore, studies conducted in Ukraine [25], France [26], and Vietnam [6] showed an even higher proportion for late onset of HIV treatment (i.e. more than 45%). The main factors associated with these high figures were: low educational attainment, non-appearance of symptoms, and not having time to go to an AIDS centre in Ukraine [25], and low levels of awareness about the disease [26], feeling healthy, and a history of intravenous drug use in Vietnam [6]. Similarly, feeling healthy, not having enough time to go to an HIV treatment centre, unwillingness to discuss HIV test results, and fear of stigma and discrimination within their community were the most frequent reasons for delayed initiation of HIV treatment in our study.

In the present study, almost half of the respondents delayed HIV treatment because they were not feeling ill. Feeling healthy and late onset of HIV-related signs and symptoms as a major factor in the delayed initiation of HIV treatment has also been observed in a number of previous studies [6, 9, 25, 27, 28]. However, 42.6% of PLHIV delayed their treatment due to not having enough time to visit the HIV clinic. Previous studies have also reported that PLHIV delay their visit to an HIV treatment centre because they have heard about long, complex, and time-consuming processes at such centres, comprising of multiple appointments, numerous physical examinations, and too much time spent waiting to consult a physician [6, 25].

The public’s negative attitudes, ideas, and viewpoints about HIV disease might explain the fact that 37.6% of our study respondents delayed their HIV treatment because they did not want to talk about their HIV-positive test result. This is even further reinforced by the fear of stigma and discrimination within their community. Our findings were similar to those of previous research in countries like Zimbabwe [29], Brazil [21], Lao People’s Democratic Republic [27], and Vietnam [6, 30, 31], where PLHIV perceived that, after starting HIV treatment, their HIV-positive result would become known to their relatives.

Our study’s findings, after applying multivariable logistic regression, revealed that rural residence and living in a district other than Lahore were significantly associated with late onset of HIV treatment. People living in rural areas and districts other than Lahore have less awareness about HIV, lower educational status, and more difficulties in visiting HIV management centres, which are situated far away from their homes, due to transport problems and cost issues [12, 32]. In developing countries like Pakistan, HIV treatment coverage is low as only 24,362 PLHIV out of 190,000 cases are receiving ART in 49 treatment centres situated in different parts of the country, particularly in big cities [33, 34]. The low healthcare coverage for HIV results in late diagnosis, poor linkages with healthcare facilities, and consequently delayed onset of ART [33, 34]. Like other developing countries, there is a need to initiate U=U campaigns (undetectable equals untransmissible) to develop awareness that PLHIV who adhere strictly to ART have undetectable level of virus in their blood and so are very unlikely to transmit it to others [7].

This study also indicates that illiteracy is also related to late commencement of HIV treatment, which is in accordance with prior studies [4, 12, 25, 27, 35]. This could be because illiterate patients have less knowledge about HIV, including risk factors, detection, prevention, management, and prognosis. They have less contact with print media, less involvement in HIV-related workshops, and less communication with other people.

Our study also found an association between CD4 count and late onset of HIV treatment. The relationship between delayed onset of HIV treatment and high CD4 count can be explained by the fact that many asymptomatic PLHIV feel themselves to be healthy. As a result, they suppose that they can delay HIV treatment until the emergence of symptoms [24]. In addition to social factors, awareness and implementation of the WHO guidelines regarding HIV treatment are very important for timely initiation of ART. Initiation of ART, irrespective of CD4 count, is not only important for reducing HIV transmission but also significant for PLHIV who have poor access to services and high loss to follow-up after diagnosis [13]. However, in Pakistan, the new guidelines are still not being followed. There is a need to adopt the WHO’s new guidelines on the timing of onset of HIV treatment [13, 14].

Furthermore, in the present study, occasional household income was related to delayed onset of ART. Similar results are seen in previous studies conducted in the USA [10, 36] and India [12]. Patients with fewer financial resources spend less money on their treatment, such as paying for medicines and regular visits to their HIV management centre. PLHIV with low socio-economic status usually seek healthcare from government hospitals. Previous research has also highlighted that PLHIV diagnosed at government testing centres or health facilities were more likely to present late for HIV care than those diagnosed at a private hospital [34]. People with low socio-economic status are less likely to receive HIV testing and care from private facilities due to the high cost, so they only engage in care when they develop symptoms [34]. Additionally, not all HIV/AIDS-related health services are available in government facilities in Pakistan and patients need to pay out of their own pockets for some services [15, 33]. Pakistan lacks a health insurance system, which has also resulted in low engagement with HIV care and late initiation of ART [34].

### Limitations and strengths

Our research findings cannot be applied to the entire Pakistani population because we only included those HIV-positive individuals who came to visit HIV clinics in selected hospitals in Lahore for HIV treatment. Our research questionnaire does not include details about factors like self-medication or the nature of a participant’s occupation, although this is presumably associated with late onset of HIV treatment. A sufficiently large sample size has been included, which helped in controlling the confounders and also identified potential risk factors for delayed onset of HIV treatment. Despite these limitations, our study has considerable strengths. Face-to-face interviews were carried out by the first author himself to collect the data, therefore an improved quality data was collected for this study.

## Conclusions

Our study results show that delayed onset of HIV treatment in Pakistan is common. Factors such as occasional household income and no schooling were significantly correlated with delayed initiation of ART. The main reasons for the late onset of HIV treatment were feeling healthy and not having symptoms, not having time to go to an HIV treatment centre, lack of willingness to discuss their HIV test result, and fear of stigma and discrimination within their community.

An integrated, multidisciplinary, comprehensive, and patient-focused approach is needed to promote the well-timed initiation of HIV treatment in order to reduce both HIV transmission and mortality. HIV management centres should counsel and monitor patients from the time of a positive HIV test result until they initiate treatment.

## Supplementary Information

**Additional file 1.** Questionnaire.

## Data Availability

The data is available from the corresponding author upon on reasonable request.

## Abbreviations

AIDS: Acquired immunodeficiency syndrome
AOR: Adjusted odds ratio
ART: Anti-retroviral treatment
CD4: Cluster of differentiation 4
CDC: Centers for Disease Control and Prevention
CI: Confidence interval
FSW: Female sex worker
HIV: Human immunodeficiency virus
IDU: Intravenous drug user
Lao PDR: Lao People’s Democratic Republic
MSW: Male sex worker
P&SHD: Primary and Secondary Healthcare Department
PACP: Punjab AIDS Control Program
PCR: Polymerase chain reaction
SD: Standard deviation
SPSS: Statistical package for social sciences
USA: United States of America
