# Supplementary material for: Frequency and reasons for delayed treatment initiation after HIV diagnosis: cross-sectional study in Lahore, Pakistan
Source: BMC Public Health. 2021 May 27;21:1000. doi: 10.1186/s12889-021-11031-0 (PMC8161554; doi:10.1186/s12889-021-11031-0)
Supplement: Supplementary file 1 — Additional file 1. Questionnaire. [file 12889_2021_11031_MOESM1_ESM.pdf]

INSTITUTE OF SOCIAL & CULTURAL STUDIES
UNIVERSITY OF THE PUNJAB

Verbal Informed Consent Format

Research Topic: Frequency and Reasons for Delayed Treatment Initiation after HIV Diagnosis: Cross-sectional Study in Lahore, Pakistan

Dear Sir/ Madam,

Assalam-o-Alaikum!

We are conducting a research to assess the frequency and reasons for delayed HIV medical care after diagnosis which will help us to plan and promote better health strategies for HIV-infected patients in order to decrease the morbidity and mortality due to delay in initiation of HIV treatment. This will take about 15 minutes of your time. Your participation is voluntary. This is an anonymous research; we will not ask about your personal details such as name, date of birth, telephone number, or address. All the information you will provide to us will be kept confidential. We request you to participate in this study. Thank you!

INSTITUTE OF SOCIAL & CULTURAL STUDIES
UNIVERSITY OF THE PUNJAB

Questionnaire

Research Topic: Research Topic: Frequency and Reasons for Delayed Treatment Initiation after HIV Diagnosis: Cross-sectional Study in Lahore, Pakistan

Serial # ____________ Hospital Registration # ____________ Date: ____________

1. Socio-demographic details of HIV-infected patients

Age: _____________ years

Gender: □ Male □ Female □ Transgender

Religion: □ Muslim □ Christian □ Other (Please specify): _____________

District of residence: _____________

Place of residence: □ Urban □ Rural

Marital status: □ Unmarried □ Married □ Separated □ Divorced □ Widowed

Educational level: □ No schooling □ 1-5 years of schooling □ 6-10 years of schooling □11-12 years of education □ University education

Employment status: □ Employed (formally or informally) □ Unemployed

Type of household income: □ Permanent □ Occasional (not regular)

2. HIV-related characteristics of HIV-infected patients

Date of confirmation of HIV diagnosis by positive HIV PCR test: ___________

Date of initiation of HIV treatment after positive HIV PCR test: ____________

Delay in initiation of HIV treatment since positive HIV PCR test: ___________

Initial CD4 count at time of initiation of HIV treatment: _________ cells/ mm^3

What was the main reason that you went for HIV testing:

□ Appearance of symptoms □ Due to HIV-positive partner

□ as part of routine follow up □ as routine test of pregnancy

□ Just wanted to know □ Other (Please specify): ______________

What do you think could be the risk factor for HIV transmission:

□ Heterosexual □ Homosexual

□ Intravenous drug use □ Other (Please specify): ______________

Do you have any physical disability: □ No □ Pre-HIV □ Post-HIV

Do you have any associated chronic disease: □ Tuberculosis □ Diabetes

□ Hypertension □ Hepatitis B □ Hepatitis C □ None

3. Now I will ask you about the reasons for delay of more than three months in initiation of HIV treatment after your positive HIV PCR test

Do you think that you felt healthy and thought the treatment wasn’t necessary	□ Yes	□ No
Due to fear of stigma and discrimination in community	□ Yes	□ No
Didn’t have time to go to HIV treatment centre	□ Yes	□ No
Fear the HIV treatment wasn’t confidential	□ Yes	□ No
Fear of medication side effects	□ Yes	□ No
Afraid of the cost/ didn’t have money	□ Yes	□ No
Fear of stigma and discrimination at facility	□ Yes	□ No
Didn’t want to take medicine for HIV	□ Yes	□ No
Didn’t know the location of HIV treatment centre	□ Yes	□ No
HIV treatment centre was too far	□ Yes	□ No
Administration and formalities were too difficult	□ Yes	□ No
Fear of detention or imprisonment	□ Yes	□ No
Perception of low service quality at facility	□ Yes	□ No
Didn’t want to think about being HIV-positive	□ Yes	□ No
Didn’t believe HIV test result	□ Yes	□ No
Didn’t want to discuss HIV result	□ Yes	□ No
Didn’t have health insurance	□ Yes	□ No
Moved out of town	□ Yes	□ No
Test not performed in the hospital providing HIV care	□ Yes	□ No

4. Knowledge regarding HIV/ AIDS among HIV-infected individuals who delayed HIV treatment more than three months after positive HIV PCR test

Heard about HIV/ AIDS	□ Yes	□ No
Healthy looking individual having HIV/ AIDS	□ Yes	□ No
HIV/ AIDS curable disease	□ Yes	□ No
HIV/ AIDS transmits by unprotected sexual contact	□ Yes	□ No
HIV/ AIDS transmits by unsafe blood transfusion	□ Yes	□ No
HIV/ AIDS transmits by needle stick injury	□ Yes	□ No
HIV/ AIDS transmits from mother to child during pregnancy	□ Yes	□ No

Thank you!
